# Facile whole mitochondrial genome resequencing from nipple aspirate fluid using MitoChip v2.0

**DOI:** 10.1186/1471-2407-8-95

**Published:** 2008-04-10

**Authors:** John P Jakupciak, Andrea Maggrah, Samantha Maragh, Jennifer Maki, Brian Reguly, Katrina Maki, Roy Wittock, Kerry Robinson, Paul D Wagner, Robert E Thayer, Ken Gehman, Teresa Gehman, Sudhir Srivastava, Alioune Ngom, Gabriel D Dakubo, Ryan L Parr

**Affiliations:** 1 Cipher Systems, Crofton, USA; 2 Genesis Genomics Inc, Thunder Bay, Canada; 3 National Institute of Standards and Technology, Gaithersburg, USA; 4 Division of Cancer Prevention, National Cancer Institute, Rockville, USA; 5 Thunder Bay Regional Health Sciences Centre, Thunder Bay, Canada; 6 School of Computer Science, University of Windsor, Windsor, Canada

## Abstract

**Background:**

Mutations in the mitochondrial genome (mtgenome) have been associated with many disorders, including breast cancer. Nipple aspirate fluid (NAF) from symptomatic women could potentially serve as a minimally invasive sample for breast cancer screening by detecting somatic mutations in this biofluid. This study is aimed at 1) demonstrating the feasibility of NAF recovery from symptomatic women, 2) examining the feasibility of sequencing the entire mitochondrial genome from NAF samples, 3) cross validation of the Human mitochondrial resequencing array 2.0 (MCv2), and 4) assessing the somatic mtDNA mutation rate in benign breast diseases as a potential tool for monitoring early somatic mutations associated with breast cancer.

**Methods:**

NAF and blood were obtained from women with symptomatic benign breast conditions, and we successfully assessed the mutation load in the entire mitochondrial genome of 19 of these women. DNA extracts from NAF were sequenced using the mitochondrial resequencing array MCv2 and by capillary electrophoresis (CE) methods as a quality comparison. Sequencing was performed independently at two institutions and the results compared. The germline mtDNA sequence determined using DNA isolated from the patient's blood (control) was compared to the mutations present in cellular mtDNA recovered from patient's NAF.

**Results:**

From the cohort of 28 women recruited for this study, NAF was successfully recovered from 23 participants (82%). Twenty two (96%) of the women produced fluids from both breasts. Twenty NAF samples and corresponding blood were chosen for this study. Except for one NAF sample, the whole mtgenome was successfully amplified using a single primer pair, or three pairs of overlapping primers. Comparison of MCv2 data from the two institutions demonstrates 99.200% concordance. Moreover, MCv2 data was 99.999% identical to CE sequencing, indicating that MCv2 is a reliable method to rapidly sequence the entire mtgenome. Four NAF samples contained somatic mutations.

**Conclusion:**

We have demonstrated that NAF is a suitable material for mtDNA sequence analysis using the rapid and reliable MCv2. Somatic mtDNA mutations present in NAF of women with benign breast diseases could potentially be used as risk factors for progression to breast cancer, but this will require a much larger study with clinical follow up.

## Background

The increased number of cancer cases around the world is a major concern. Research methods for identifying the presence of cancerous cells by measuring mutations in mtDNA is the subject of intense clinical investigation [1,2]. Frequently, these studies analyze only specific regions of mtDNA and not the entire mitochondrial genome (mtgenome). There are several biological characteristics of mitochondria, and in particular the mtgenome that make it suitable for early detection and monitoring of neoplasia. This genome has an accelerated mutation rate in comparison to the nuclear genome and accrues somatic mutations in tumor tissues [2-5]. The mitochondrial genome has a high copy number in comparison to the nuclear archive of DNA; there are potentially 1,000s of mtgenomes per cell, which enables easy detection of important biomarkers, even when only low amounts of samples are available.

Given its small size and vital role in bioenergetics, the mtgenome is frequently sequenced in its entirety [1,2,6]. MtDNA mutations increase the risk of mitochondrial associated diseases later in life [7]. Here we report the feasibility of sequencing the complete mtgenome from nipple aspirate fluid (NAF), and the reliability of microarray based resequencing of the mtgenome to CE sequencing technology as well as the reproducibility of the resequencing methodology in a cross validation study between the National Institute of Standards and Technology (Gaithersburg, MD USA) and Genesis Genomics Inc. (Thunder Bay, On Canada). This study also uncovered mtgenome mutations in NAF, a relatively easily obtained body fluid, which has potential use for early breast cancer detection, diagnosis and monitoring [8].

Dr. Susan Love pioneered the intraductal approach to access the lobular units where breast cancer begins [9]. NAF can be recovered from 48% to 94% of women and represents a minimally invasive technique for obtaining breast fluids for the evaluation of abnormalities associated with breast cancer [10-14]. NAF production has been linked to an increased relative risk for breast cancer development [15], and the evaluation of NAF as a potential screening tool for the detection of breast cancer has been the subject of intense investigation [16-18]. NAF from women with no clinical evidence of breast tumors has also been analyzed to detect mtDNA mutations and for genetic variants (BRACA1) that are associated with breast cancer development [19].

A limitation of using NAF for diagnosis has been the sensitivity of detection technologies. However, with recently developed technologies and modern sensitive cancer biomarkers [20], the usefulness of NAF deserves re-evaluation. An important limitation of the use of NAF is its low cellular content, which often precludes histopathologic analyses [12]; however, analyses of predictive markers in this biofluid can identify the presence of cancer [21]. Ductal lavage is a technique designed to overcome low cellularity and allows access to an entire duct system. This procedure results in high cytological/histological specificity (100%), but low sensitivity and accuracy (17% and 19% respectively) [22]. The analysis of NAF to monitor recurrence may have limited application, because risk reduction therapies, including selective estrogen receptor modulators (tamoxifen, raloxifene or oophorectomy), appear to reduce NAF yields [15].

The high copy number of the mtgenomes [23] is an advantage of using mtDNA SNP analysis for early detection and monitoring disease progression. In addition, rapid, accurate and relatively low cost resequencing methods streamline mutation detection and justify complete mtgenome analysis of cancer associated mutations in tumor and preneoplastic tissues. Sequencing the entire mtgenome allows for a complete inventory of the point mutations in this genome that may develop in association with disease pathology and provide early detection markers [1].

Herein we report the utility of NAF for rapid whole mtgenome analysis. This study represents the largest number of NAF samples sequenced, and the first application of DNA microarray measurement of the mutation load in NAF. The results were independently cross-validated. Microarray analysis has an increased sensitivity over fluorescent sequencing [24]. We detected somatic mutations in NAF that were not present in the matched control tissue. While our study does not answer the etiology behind mutations present in NAF, our findings suggest that this paradigm may be useful for screening NAF for mtDNA mutations analogous to recent clinical proposals [25].

## Methods

### Study Subjects

Symptomatic women referred to a surgical oncologist for a clinical breast examination and who had a negative result were recruited for this study. A total of 20 women were selected for the cross validation study. All patients were recruited in accordance with the ethical guidelines of the Thunder Bay Regional Health Sciences Ethics Board in adherence to the Tri-Council Policy Statement on Ethical Conduct for Research Involving Humans. Written consent was obtained from the patients for publication of the study.

### Samples

Blood samples were collected by standard venipuncture using a BD Vacutainer CPT™. Additionally, blood from a finger prick was collected on isocode cards from each patient. MtDNA was extracted from whole blood using a QiaAmp DNA MiniKit (Qiagen). NAF was recovered by a qualified practitioner, using a FIRSTCYTE™ Aspirator (Cytyc Health Corporation) following the recommendations of the manufacturer, and stored in CytoLyt Solution until extracted. Using this device, NAF was expressed from 1–3 ducts and pooled for the study. This method of NAF collection is therefore not representative of the entire ductal system, and could miss ducts with lesions. For diagnostic purposes, a better method of NAF collection is needed. The total volume of NAF collected per patient ranged from 50 – 100 μL. MtDNA was extracted using the QiaAmp DNA MiniKit (Qiagen) and archived at -86°C. Both NAF and blood isolated mtDNA samples were randomized and blinded for the cross-lab validation.

### Mitochondrial genome amplification

#### Method used at Genesis Genomics Inc

The entire mtgenome of both NAF and corresponding blood samples were amplified using a single back-to-back primer set or three overlapping primers (see primer sequences in Table 1). These primers were designed using proprietary software that precludes pseudogene coamplification. 25 ng of template DNA, 5U LA Taq polymerase (TaKara), 5 μL buffer, 2.5 mM each of dNTPs, 0.2 μM of primers were mixed with dH_2_0 to a final reaction volume of 50 μL. Cycling parameters were as follows: 94°C for 1 min, followed by 35 cycles of 94°C for 10s, 68°C for 15 min, 72°C for 10 min, and a final hold at 4°C.

**Table 1:** Primers used for whole mtgenome amplification at GGI

**Primer name**	**Mtgenome location**	**Primer sequence 5' – 3'**
Mt12s long R	1135	ccagaacactacgagccacag
Mt12s long F	1076	gtgttatcccagtttgggtcttagcta
617F	617	gtttagacgggctcacatcacc
6027R	6027	cagctcggctcgaataaggag
5819F	5819	tcggagctggtaaaaagaggcctaac
11783R	11783	gatgcgactgtgagtgcgttcgtag
11268F	11268	ccctaggctcactaaacattctac
731R	731	tagagggtgaactcactggaa

#### Method used at NIST

DNA amplification was performed independently at NIST using three primer pairs previously used for fluorescent DNA sequencing resulting in 3 amplicons of 5–6 kb in length for full coverage of the mitochondrial genome [26]. Samples which did not amplify using these primers were successfully amplified using the nine primer sets previously validated for full mtDNA fluorescent sequencing [2]. Each PCR product was visualized on an agarose gel to obtain a qualitative assessment of the amount of mtDNA generated by the procedure. The mitochondrial DNA template, 10 μmol of primers, 0.5U LA Taq polymerase (TaKara), 5 μL buffer, 8 μL dNTPs (10 μmol each) and 33 μL of dH_2_0 were mixed for a total reaction volume of 50 μL. Thermal cycling conditions were as follows: 94°C for 2 min, followed by 30 cycles of 94°C for 15 s, 68°C for 7 min; final elongation 68°C for 12 min; 4°C hold. PCR amplification products were analyzed for quality and quantity as previously [2] or by spectrophotometric methods as described in GeneChip CustomSeq™ Resequencing Array Protocol Version 2.

### PCR Cleanup: MitoChip

PCR clean up was conducted using the QIAquick 96 well vacuum plate manifold and protocol (Qiagen). DNAs were eluted into 65 uL of DNAse/RNAse free water.

### CE-based Fluorescent Sequencing

Amplified mtgenome template was sent to CoGenics (Houston, Texas) for capillary electrophoresis sequencing. Briefly, Big Dye Termination Chemistry and a series of 72 primers were used to sequence in both forward and reverse directions. The fluorescent PCR-based sequencing was also conducted independently as a cross validation at NIST using previously published reaction conditions [2]. Primers contained M13 tags to facilitate DNA sequencing with M13 forward and reverse sequences. Briefly, the blood and NAF mtDNAs were sequenced using the Big Dye™ Terminator (BDT) version 3.1 cycle sequencing kit (ABI). A one eighth cycle sequencing reaction was used for each primer. Reactions contained 1 μL of each of the following reagents: BDT reagent, DNA (3–6 ng/μL), M13 primer (forward or reverse; 5 pmol/μL), 5× Dilution Buffer (ABI), and dH_2_O to a final volume of 5 μL. Cycling sequencing conditions for forward primers were as follows: (40 cycles): 96°C for 10 s; annealing, 50°C for 5 s; elongation, 60°C for 4 min; 4°C hold. Reverse primers were sequenced using the same protocol, but the annealing temperature was lowered to 37°C.

The Montage™ SEQ_96 _plate (Millipore Corp., Billerica, MA) was used for clean-up following the cycle sequencing reactions. Thirty microliters of Wash Solution was added to each well of the cycle sequencing plate. The samples were transferred to the clean-up plate and placed on the vacuum manifold for 15–20 minutes or until the wells were dry. A second wash of 30 μL Wash Solution was added and vacuumed dry for an additional 25 to 30 minutes. Once dry, 20 μL of Injection Solution were added to each well and the plate was mixed vigorously on a plate shaker for 10 minutes. Resuspended samples were transferred to a 3100 Optical Plate and diluted with 15 μL of HI-DI Formamide (ABI). All separations were performed using the ABI 3130×LGenetic Analyzer with an 80 cm capillary and POP7 polymer system. Samples were electrokinetically injected (30 seconds, 1 KV) and separated at 14.6 KV. Sequences were aligned using the DNA Star SeqMan II (5.05) program and scanned for polymorphisms and sequence variants in direct comparison to revised Cambridge Reference Sequence (rCRS) and the corresponding sequence of the blood.

### Resequencing: MitoChip protocol

The GeneChip^® ^CustomSeq^® ^Resquencing Array Protocol Version 2 was used with a few modifications. Following amplification of the mtgenome, template was prepared and hybridized as recommended by the GeneChip^® ^Resequencing Reagent Kit. MCv2 chips were processed in the GeneChip^® ^Hybridization Oven 640, GeneChip^® ^Fluidics Station 450 and the GeneChip^® ^Scanner 3000. Briefly, either three or nine amplicons representing the patient and normal control mitochondrial genomes were separately pooled at equi-molar concentrations. The PCR amplification products were pooled, fragmented, labeled, hybridized, washed, and scanned. The total quantity of DNA applied to the array was 0.62 μg. Fragmentation of the pooled DNAs was conducted using 0.15 units of Fragmentation reagent (0.033 μL) per sample at 37°Cfor 15 minutes followed by 95°C for 15 minutes to inactivate. The fragments were labeled with 30 units of TdT at 37°C for 90 minutes followed by 95°C for 15 minutes. The hybridization cocktail, including separately prepared control fragments, was hybridized for 16 to 18 hours at 45°C with 60 rpm. Arrays were washed using the mini_mapping10kv1_450 fluidics protocol, scanned on a GeneArray^® ^2500 Scanner or a GeneChip^® ^Scanner 3000G7 Scanner, and analyzed with GeneChip^® ^DNA analysis (GDAS) and GSEQ Softwares.

### MitoChip Sequence Interpretation

Final analysis of all data was conducted using Affymetrix software GCOS v1.4 and GSEQ v4.0. The probe intensities for each mutation reported by the software were examined on the forward and reverse strands for every occurrence of a specific base position located on the chip. Mutations were confirmed and only reported when the mutation was seen on both strands for locations appearing once, and 3 of 4 strands for locations that appear on the chip twice. We selected a random subset of our patient samples (3 tissues each from 4 patients) to evaluate the accuracy of the MitoChip in comparison to CE DNA sequencing. All samples were blinded and phylogenetic trees were returned using the PHYLIP program Dnaml, Dnapars and Dnadist [27].

### Short Tandem Repeat (STR) Genotyping

All samples were genotyped using the PowerPlex^® ^16 System (Promega Corp, Madison, WI) on a 3130×L genetic analyzer with a 36 cm capillary array and POP4 polymer and analyzed using GeneMapper^® ^*ID *v3.2 (Applied Biosystems, Foster City, CA (ABI). Samples were diluted to 0.5 – 1.0 ng/μL and 1 μL of sample was added to a 24 μL reaction volume (18.2 μL H_2_O, 2.5 μL 10× buffer, 2.5 μL PowerPlex^® ^16 10× primer pair mix, 0.8 μL (4U) AmpliTaq Gold^® ^DNA Polymerase (ABI), then PCR amplified using published conditions. 1 μL of ILS600 internal lane standard and 9 μL of HiDi™ Formamide (ABI) were added to 1 μL of reaction (or 1 μL Allelic Ladder Mix, one for each run) then the mix was briefly denatured and chilled to 95°C and then placed on crushed ice for 3 minutes prior to each sequencing run.

## Results and Discussion

### Quality assurance

STR typing was performed on all samples to eliminate the possibility of inadvertent sample mix-up or cross contamination.

### Sequencing of whole mtgenome from NAF samples

Two previous studies have examined mtDNA mutations in NAF, however, both studies examined less than 30% of the mtgenome [18,19]. For diagnostic purposes, it is more informative to interrogate the complete mtgenome. Hence, our attempt was to reliably sequence the complete mtgenome from NAF. NAF was successfully extracted from 23 of the 28 women recruited for this study. Twenty two (96%) of the women produced NAF from both breasts, and 20 NAF samples from the left breasts and matched blood were chosen for this study. Table 2 shows the clinicopathologic and demographic data of the study participants. Overall thirty-nine (20 blood and 19 NAF) samples were successfully processed; one NAF sample could not be fully amplified and was not included in the analysis. The entire mtgenome of 14 out of 19 NAF samples were successfully amplified using a single full length primer pair, with the remaining 5 samples requiring three overlapping primers. Although the primers were designed to preclude pseudogene co-amplification, the ability to amplify the full length mtgenome in biological samples such as NAF with reduced cellularity is an added quality assurance measure against pseudogenes. Sequence comparison between the NAF, blood and rCRS revealed a total of 490 polymorphisms and 7 mutations. Of the 7 mutations (SNPs discovered only in the NAF and not present in the blood) noted between all samples, three were associated with known problematic features of MCv2 at nucleotide positions 9179, 9914, and 11719 (Table 3). Comparison of MCv2 sequence data to the gold standard (CE) revealed a 99.999% identity. These results indicate that microarray resequencing of the mtgenome recovered from NAF is a rapid, sensitive and cost effective technique. Importantly, this technology enables clinical access to the information content of the mtgenome of mammary ductal epithelial cells.

**Table 2:** Clinicopathologic and demographic characteristics of study participants

**Patient ID**	**Age (years)**	**Family history of breast cancer**	**Breast findings at examination**
1059	42		Right breast cyst; Aspiration biopsy
1069	51		Normal
1070	59		Normal
1071	61	Aunt	Nipple discharge; Mastitis
1086	50		Large cyst
1087	51	Aunt	Small solid hypoechoic nodule
1126	44	Grandmother	Bilateral dense/nodular breast; Cyst in left breast
1135	40		Cystic breast masses
1139	39		Normal
1140	56		Cyst
1165	53	Two aunts	Normal
1178	35		Galactocele
1179	36	Mother	Normal
1180*	56		Normal
1181	36	Mother and grandmother	Benign lump
1182	43		Normal
1184	53	Mother	Right breast mass; FNA negative for malignancy
1185	50		Fibrocystic lesions; Previous breast biopsy
1192	48	Mother and aunt	Normal
1193	41		Microcalcification

*NAF from this patient failed to amplify

**Table 3:** Comparative analysis of NIST and GGI sequence data

**Number of call differences between all samples**	**Concordance (%)**	**Sequence Identity (%)**
7 total	98.592	99.999
3/7 associated with problematic features: 9179, 9914, and 11719	99.190	99.999
4/7 heteroplasmic	100.000	100.000

### Cross validation of MCv2

**Figure 1:**
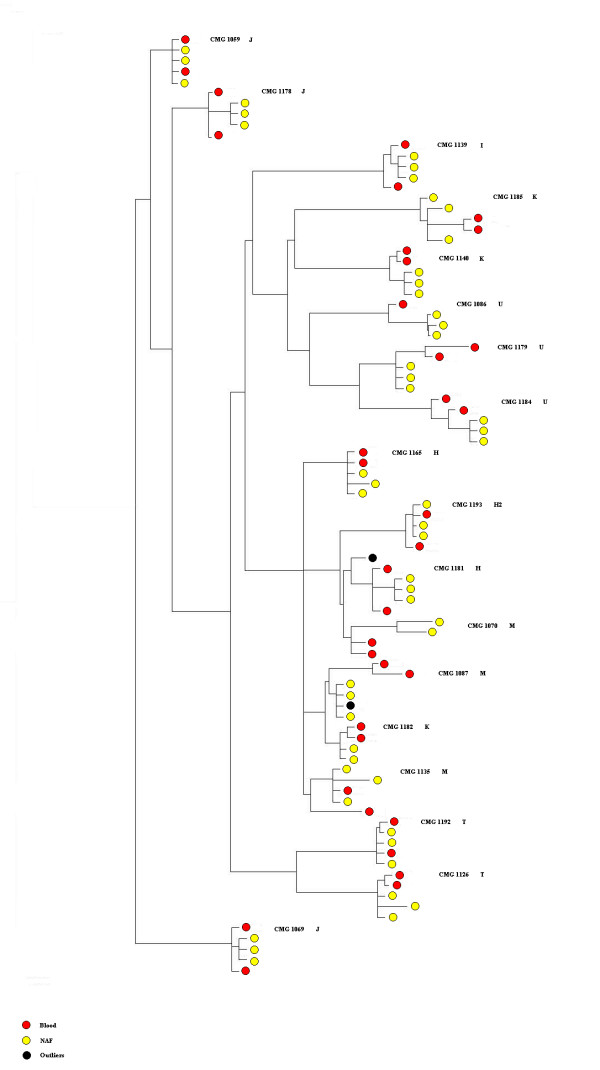
**Maximum likelihood tree showing the relationship between individual patients' mtgenomes derived from both blood and NAF.** There are two independent sequences for each blood sample (MCv2 and CE; red circles), and three independent sequences for each NAF sample (MCv2 sequences from GGI and NIST, and CE sequences; yellow circles). Results are also clustered according to haplogroups. Individual CMG1182 has several identical polymorphisms to haplogroup M and therefore clusters with this group even though she is haplogroup K. Individual CMG1071 was excluded from this analysis because the three NAF sequences had several nucleotide differences. Black circles represent outlier sequences.

The ability to rapidly sequence the mtgenome from NAF was cross-lab validated by independent confirmation at separate venues with a 99.200% concordance. Table 3 records the comparative data between the work done at NIST and Genesis Genomics, demonstrating repeatability of the detection method and consensus on the number of mtDNA mutations. Maximum likelihood clustering analysis of the blinded results from both labs demonstrates general clustering from the suite of samples from any given subject (Figure 1), again demonstrating the accuracy of the data obtained at each institution. Moreover, each individual was resolved into mitochondrial haplogroups, as expected. Haplogroup polymorphisms were accurately detected in both NAF and blood samples from the respective participant. In general, this study population was essentially of European ancestry. Although Figure 1 appears to indicate differences between the NAF and blood samples from a given patient, this effect is an artifact since the genetic distances between these samples is very small (e.g. 0.00001). These small distances are also true for the general outline of the tree.

### Elevated somatic mtDNA mutations in benign breast disease

Four out of 19 (21%) NAF samples contained a single point mutation difference from the matched controls (blood) (Table 4). Previous work on partial mtgenome analysis is consistent with our results [18]. This work by Zhu *et al*. reported that some mutations detected in NAF were also found in the patient's primary tumor, emphasizing the possible utility of NAF for breast cancer screening. Although no tumor was detected in our cohort of women, these mutations may indicate mtgenome instability, perhaps suggesting an elevated risk for breast cancer in these 4 women. For example, patient 1069 had a C/T heteroplasmy at bp 516, which is within a D-Loop region of known mtgenome instability in breast tumors [28]. Heteroplasmy is considered an early marker of potential disease [29,30]. Moreover, one study concluded that breast tumors that have mutations within the D-Loop have less favorable patient outcomes than those lacking these alterations [31]. Thus this simple, sensitive measurement of the mutation load from non-invasively collected samples, offers an alternative for diagnostic purposes.

**Table 4:** Somatic mutations identified in patients symptomatic of breast pathology

**Sample**	**Pathology**	**Mutation in NAF**	**Locus**
1069	Normal	C516Y	C/R D-loop
1086	Large cyst	A4188G	ND1
1135	Cystic masses	T6776C	COI
1139	Normal	G1320R	12S rRNA

One important aspect of this project is the massive amount of genetic data collected in a relatively short period. Close to 1.6 × 10^6 ^mtgenome bases were sequenced, demonstrating an extremely fine genetic resolution when using the MCv2. This suggests that cross-validated point mutations may indeed be important indicators of altered molecular processes indicative of potential transformation. Independent cross-validation confirmed these mutations. Combined with the broad resolution demonstrated by the blinded Maximum likelihood clustering results, in association with corresponding demographic data, this technology is capable of gathering a broad spectrum of population level data. Moreover, this methodology may have important utility in the early detection of breast cancer associated mutations in NAF.

## Conclusion

Ductal lavage and random periareolar fine needle aspiration are both used to harvest breast epithelial cells for risk assessment as well as to evaluate response in chemoprevention trials. The magnitude of increase in relative risk has been defined and other studies have concluded that presence of NAF with epithelial cells is associated with an increase in breast cancer risk and that such cells harbor useful markers for women at higher risk [32]. However, the evaluation of NAF has shown limited promise in part, because of poor cytologic reproducibility [33]. The measurement of cancer associated mutations in the entire mtgenome may overcome such limitations.

Resequencing with microarray technology is a rapid, highly accurate, relatively inexpensive method, which enables the widespread investigation of mitochondrial mutations detected in non-invasively collected body fluids associated with solid tumors. The cost saving benefits of chip-based sequencing techniques, including reagents, labor, time-to-results, ease and accuracy of data interpretation, are substantial in comparison to typical fluorescent sequencing methods. For example, 12 mtgenomes can be sequenced with a chip per day, as opposed to 12 mtgenomes per month, using a 16 capillary DNA sequencer, a 30-fold increase in productivity and data acquisition. This demonstrates potential, important utility from a disease detection perspective. Finally, the chip can detect low-level heteroplasmy, a condition often associated with the genesis of disease.

## Abbreviations

NAF, nipple aspirate fluid; MCv2, MitoChip v2.0; CE, capillary electrophoresis; mtDNA, mitochondrial DNA.

## Competing interests

The author(s) declare that they have no competing interests.

## Authors' contributions

AM, SM, JM, and KR conducted experiments and helped analyze the data. BR performed sequence analysis. RW coordinated sample collection. KG and TG collected NAF samples. PW and SS helped in data analysis and preparation of the manuscript. AN performed sequence analysis and generated the Maximum likelihood tree. RET, GDD, JPJ and RLP coordinated and supervised the research, and wrote the manuscript.

## Pre-publication history

The pre-publication history for this paper can be accessed here:


